# Cysteinyl Leukotrienes Pathway Genes, Atopic Asthma and Drug Response: From Population Isolates to Large Genome-Wide Association Studies

**DOI:** 10.3389/fphar.2016.00299

**Published:** 2016-12-01

**Authors:** Miles D. Thompson, Valerie Capra, Mark T. Clunes, G. E. Rovati, Jana Stankova, Mary C. Maj, David L. Duffy

**Affiliations:** ^1^Biochemical Genetics and Metabolomics Laboratory, Department of Pediatrics, University of California, San Diego, La JollaCA, USA; ^2^Department of Laboratory Medicine and Pathobiology, University of Toronto, Toronto, ONCanada; ^3^Department of Health Sciences, San Paolo Hospital, Università degli Studi di MilanoMilano, Italy; ^4^Department of Physiology/Neuroscience, School of Medicine, Saint George’s UniversitySaint George’s, Grenada; ^5^Department of Pharmacological and Biomolecular Sciences, Università degli Studi di MilanoMilano, Italy; ^6^Division of Immunology and Allergy, Department of Pediatrics, Faculty of Medicine and Health Sciences, Université de Sherbrooke, SherbrookeQC, Canada; ^7^Department of Biochemistry, School of Medicine, Saint George’s UniversitySaint George’s, Grenada; ^8^QIMR Berghofer Medical Research Institute, HerstonQLD, Australia

**Keywords:** montelukast, pharmacogenetics, pharmacogenomics, cysteinyl leukotriene 1 (CYSLTR1), cysteinyl leukotriene 2 (CYSLTR2), epistasis, epigenetics, Tristan da Cunha

## Abstract

Genetic variants associated with asthma pathogenesis and altered response to drug therapy are discussed. Many studies implicate polymorphisms in genes encoding the enzymes responsible for leukotriene synthesis and intracellular signaling through activation of seven transmembrane domain receptors, such as the cysteinyl leukotriene 1 (CYSLTR1) and 2 (CYSLTR2) receptors. The leukotrienes are polyunsaturated lipoxygenated eicosatetraenoic acids that exhibit a wide range of pharmacological and physiological actions. Of the three enzymes involved in the formation of the leukotrienes, arachidonate 5 lipoxygenase 5 (ALOX5), leukotriene C4 synthase (LTC4S), and leukotriene hydrolase (LTA4H) are all polymorphic. These polymorphisms often result in variable production of the CysLTs (LTC4, LTD4, and LTE4) and LTB4. Variable number tandem repeat sequences located in the Sp1-binding motif within the promotor region of the *ALOX5* gene are associated with leukotriene burden and bronchoconstriction independent of asthma risk. A 444A > C SNP polymorphism in the *LTC4S* gene, encoding an enzyme required for the formation of a glutathione adduct at the C-6 position of the arachidonic acid backbone, is associated with severe asthma and altered response to the CYSLTR1 receptor antagonist zafirlukast. Genetic variability in the CysLT pathway may contribute additively or synergistically to altered drug responses. The 601 A > G variant of the *CYSLTR2* gene, encoding the Met201Val *CYSLTR2* receptor variant, is associated with atopic asthma in the general European population, where it is present at a frequency of ∼2.6%. The variant was originally found in the founder population of Tristan da Cunha, a remote island in the South Atlantic, in which the prevalence of atopy is approximately 45% and the prevalence of asthma is 36%. *In vitro* work showed that the atopy-associated Met201Val variant was inactivating with respect to ligand binding, Ca^2+^ flux and inositol phosphate generation. In addition, the *CYSLTR1* gene, located at Xq13-21.1, has been associated with atopic asthma. The activating Gly300Ser CYSLTR1 variant is discussed. In addition to genetic loci, risk for asthma may be influenced by environmental factors such as smoking. The contribution of CysLT pathway gene sequence variants to atopic asthma is discussed in the context of other genes and environmental influences known to influence asthma.

## Introduction

Given that the prevalence of asthma may be increasing in many populations world-wide (Eder et al., 2006), it is significant that advances in treatment may result from an improved understanding of both the molecular basis of asthma and its responsiveness to therapy. Both asthma susceptibility loci, the genetic variability that confers risk for complex respiratory traits that lead to asthma, and pharmacogenetics, the genetic variability that confers responsiveness of an individual to pharmaceutical therapy, may be best modeled as independent complex traits with both genetic and environmental (GXE) contributions. Since variability in the genes encoding the proteins essential for inflammation may influence risk for many aspects of asthmatic inflammation (Drazen et al., 2000), it is to be expected that it might also increase the susceptibility to asthma independent of their pharmacogenetic relevance (Thompson et al., 2008). Here, we discuss the relative contribution of genes in the cysteinyl leukotriene (CysLT) pathway to both the etiology of asthma itself and a person’s responsiveness to leukotriene modifying (LTM) drugs.

While in some cases the genetic variants that result in disease also encode protein targets for pharmaceutical interventions, this relationship is often not consistent even with polymorphisms of a G protein-coupled receptor (GPCR) system such as the one that mediates CysLT inflammation. For example, while variability in the genes encoding drug targets such as the cysteinyl leukotriene 1 (*CYSLTR1*) and 2 (*CYSLTR2*) receptor genes may both contribute to risk for atopic asthma in some populations (Thompson et al., 2003, 2005b, 2013; Pillai et al., 2004; Brochu-Bourque et al., 2011; Yaddaden et al., 2016), only the cysteinyl leukotriene 1 (CYSLTR1) receptor is targeted by LTMs. We can only speculate, however, on the role that that variability in the cysteinyl leukotriene 2 (CYSLTR2) receptor may have in LTM pharmacogenetics since drugs targeting the CYSLTR2 receptor are not widely available.

In general, respiratory disease itself, and patient response to therapy, may be best modeled as independent complex traits with both genetic and environmental contributions. The GPCR associated with asthma, the (*NPSR1*) neuropeptide S receptor 1 (Laitinen et al., 2004; Kormann et al., 2005; Melén et al., 2005; Pietras et al., 2011), may represent an exception inasmuch as it also defines a potential therapeutic target. As a result, many advances in pharmacogenetics have resulted not from linkage or candidate gene studies but from Genome Wide Association Studies (GWAS). Hence, the field of pharmacogenomics has become axiomatic in the study of patient response and non-response to medication, adverse drug reactions, and optimizing drug dose for the individual.

**FIGURE 1:**
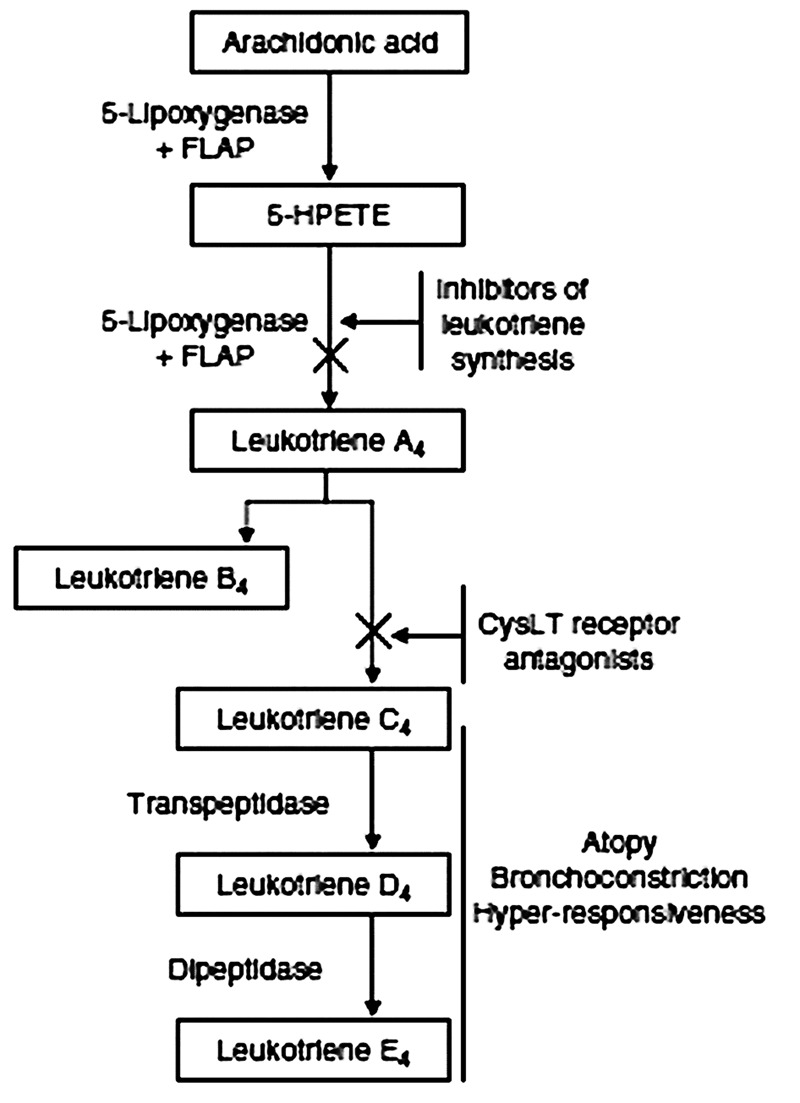
**Cysteinyl leukotriene synthesis pathway.** Leukotriene biosynthesis inhibitors act on either 5-lipoxygenase or its activating protein (FLAP). The class of cysteinyl leukotriene (CysLT) receptor antagonists target the CysLT receptors CYSLTR1 and CYSLTR2. 5-HPETE, 5-hydroxyperoxy-eiocosatetraenoic acid.

The study of *CYSLTR1* and *CYSLTR2* gene variability in asthma, therefore, must be considered in the context of the genetic polymorphisms found within the synthesis pathway genes as well as genes that modify patient response to LTMs. The LTMs used to treat asthma, such as montelukast, a drug which targets cysteinyl leukotriene 1 (CYSLTR1) receptor protein, have a greater than 20% non-response rate (Noonan et al., 1998). While some of the pharmacogenetic determinants of LTM response are attributable to variability in genes that are integral (Kang et al., 2011; Mougey et al., 2013) to the signaling of cysteinyl leukotrienes (**Figure 1**), many true pharmacogenetic variants are located in genes that lie directly outside the pathway (Dahlin et al., 2015, 2016).

In order to undertake GWAS studies of complex traits, a large sample size is generally required. Thus, informative studies of the genetic basis of drug response are conducted among large samples of unrelated individuals recruited from a given population. This is especially true for asthma phenotypes studied in out-bred populations since they tend to be genetically heterogeneous (Moffatt et al., 2007; Michel et al., 2010; Sleiman et al., 2010; Ferreira et al., 2011; Melén et al., 2013). By contrast, small candidate gene studies have achieved reasonable power for detecting variants that confer risk (Marshall et al., 2013). Since data from sequencing large patient cohorts has become available, it has been possible to assess the wider relevance of some observations originally made in smaller studies.

For example, our study of atopic asthma in the Tristan da Cunha isolate provided early insight into CysLT pharmacogenetics (Thompson et al., 2006; Capra et al., 2007; Brochu-Bourque et al., 2011). In particular, the hypomorphic Met201Val CYSLTR2 variant identified on Tristan da Cunha (Thompson et al., 2003) has since been associated with atopy in large out-bred populations (Pillai et al., 2004). Selected examples of GPCR variants associated with asthma phenotypes are presented in **Table 1**. **Tables 2** and **3** present selected variants of the *CYSLTR1* and *CYSLTR2* genes.

**Table 1:** Human G-protein-coupled receptor (GPCR) variants associated with asthma endophenotype or altered pharmacology.

Receptor (gene name)	Variant/allele	Disease/phenotype	Pharmacology	Reference
Endothelin-1 receptor type A (*EDNRA*)	A*fi* II	Atopy; IgE levels increased	Altered receptor expression	Mao et al., 1999; Michel et al., 2010
Beta2-adrenergic receptor (*ADRB2*)	R16 > G	Reduced lung function; nocturnal asthma/severity	Downregulation enhanced; albuterol response decreased	Hawkins et al., 2008; Basu et al., 2009
	Q27 > E	Hypertension risk; obesity; IgE levels increased	Downregulation resistant; albuterol response increased; drug hypersensitivity	Reihsaus et al., 1993; Dewar et al., 1997; Drysdale et al., 2000
	G16/E27 haplotype	Asthma, heart disease, immune disorders		Drysdale et al., 2000
	C341 > G	None known	Uncoupling from Gs/adenylyl cyclase system	Lachance et al., 1999
Prostaglandin D2 receptor (*PTGDR*)	Promoter SNPs undermine normal helper T anti-inflammatory response	Asthma	Altered receptor expression anti- immune response to PGD2	Cookson and Moffatt, 2004; Oguma et al., 2004
Chemoattractant receptor on Th2 cells (*CRTH2*/*GPR44*)	3′ UTR SNPs: 1651G > A, 1544C/1651A	Asthma	Altered receptor expression; inflammatory response to PGD2	Hirai et al., 2001; Wang et al., 2009
Thromboxane A2 receptor (*TBXA2R*)	TP-alpha and TP-beta isoforms SNPs	Asthma, atopy and aspirin-intolerant asthma		Unoki et al., 2000; Kim et al., 2005
Cysteinyl leukotriene receptor 1 (*CYSLTR1*)	G300S	Atopy	Receptor activation	Thompson et al., 2007; Yaddaden et al., 2016
Cysteinyl leukotriene receptor 2 (*CYSLTR2*)	M201V	Various populations	Receptor inactivation	Thompson et al., 2003, 2005b; Pillai et al., 2004; Brochu-Bourque et al., 2011
Prostaglandin D2 receptor (PTGDR)	-441T/C	No disease association	Montelukast response	Kang et al., 2011
Chemokine [C-C motif] receptor 5 (*CCR5*)	32 bp deletion; 59029 A > G	Risk of asthma decreased	Diminished CCR5 expression on Th1 cells results in a preponderance of Th2 cells; decreased binding to activity; decreased binding to CC	Hall et al., 1999; Srivastava et al., 2003; Agrawal and Bharadwaj, 2005
G-protein-coupled receptor associated with asthma (*GPRA* or *GPSR154*)	GPRA-B isoform over-expressed in bronchial epithelia of asthmatics	Asthma	Altered RANTES, MIP-1-alpha ligand suggests that GPRA is a potential drug target	Laitinen et al., 2004; Kormann et al., 2005; Melén et al., 2005; Pietras et al., 2011

*IgE, immunoglobulin E; MIP-1alpha, macrophage inhibitory protein; PGD2, prostaglandin D2; RANTES, regulated on activation, normal T cell expressed and secreted; SNP, single nucleotide polymorphism; UTR, untranslated region.*

**Table 2:** CYSLTR1 receptor variants: naturally occurring polymorphisms and *in vitro* engineered mutants.

Variant, SNP or access code)	Type	SNP access code	Disease association	Reference
c.-945T/C c.-786C/A c.-647G/A	Promotor Promotor Promotor	rs321029 rs2637204 rs2806489	Associated with IgE /atopy Aspirin intolerant asthma (AIA) associated Not associated with IgE; Associated with atopy in females	Kim et al., 2007 Zhang et al., 2006 Duroudier et al., 2009 Kim et al., 2006
TCG (above) c.-566C/T c. 927T/C	Haplotype Promotor synonymous Various haploblock	rs7066737 rs320995	Upregulating haplotype not associated Not associated Not associated Associated with asthma Not associated with atopic asthma Atopy severity in females Not associated	Zhang et al., 2006 Duroudier et al., 2009 Choi et al., 2004 Sanz et al., 2006 Kadry et al., 2014 Arriba-Mendez et al., 2006 Hao et al., 2006 Al-Shemari et al., 2008
c.898 G/A	C-terminus		300Ser is a hypermorphic variant; Increased Ca^2+^ flux	Thompson et al., 2007
			Normal expression and desensitization Increased LTD4 binding; Increased Inositol Phosphate	Yaddaden et al., 2016

*aa, amino acid; IC, intracellular loop; SNP, single nucleotide polymorphism; TM, transmebrane helix. Approximate allele frequency as reported by the Exome Aggregation Consortium (ExAC), Cambridge, MA, USA (http://exac.broadinstitute.org).*

**Table 3:** CYSLTR2 receptor variants: naturally occurring polymorphisms and *in vitro* engineered mutants.

Nucleotide position	Type	SNP access code	Disease association	Reference
g.-1220A/C c.-819 T > G c.2078 C > T c.2534A > G c.3105 A/G (Exon1-UTR) c.-2534A > G c.601 A/G	Promotor Haplotype Haplotype Coding	rs1323552 rs912278 rs41347648	Associated with asthma Aspirin intolerant asthma Stronger association with LTC4S c.-444A > C Not associated with atopic dermatitis Not associated 201Ser is associated with atopic asthma 201Ser is a hypomorphic variant; decreased Ca^2+^ flux Common allele associated with protection against atopy Associated with atopy; Hypomorphic 201Ser is hypomorphic Decreased LTD4 binding Decreased inositol phosphate production	Fukai et al., 2004 Park et al., 2005 Kato et al., 2011 Kumar et al., 2012 Thompson et al., 2003 Thompson et al., 2006 Pillai et al., 2004 Brochu-Bourque et al., 2011

*aa, amino acid; IC, intracellular loop; SNP, single nucleotide polymorphism; TM, transmebrane helix. Approximate allele frequency as reported by the Exome Aggregation Consortium (ExAC), Cambridge, MA, USA (http://exac.broadinstitute.org).*

### Population Isolates

Studies of population isolates rely upon the fact that a founder effect limits genetic heterogeneity and results in greatly increased prevalence rates (Roberts, 1968; Zamel et al., 1996). In the Tristan da Cunha isolate, for example, there is a 45% prevalence of atopy and a 36% prevalence of asthma (Mantle and Tyrrell, 1973; Zamel et al., 1996; Camacho et al., 2011; Camacho and Cazelles, 2013). By comparison, the prevalence of asthma in most out-bred populations rarely exceeds 10%. The severity of the Tristan da Cunha atopy phenotype may be reflected in events such as the Tristan da Cunha influenza epidemic of 1971 (Mantle and Tyrrell, 1973; Zamel et al., 1996; Camacho et al., 2011; Camacho and Cazelles, 2013).

Analyzing the transmission of genetic markers in a large extended pedigree such as a population isolates, provides the opportunity to identify risk alleles originating from common ancestry (Soodyall et al., 1997, 2003). In particular, this enrichment optimizes the analysis of complex traits for gene-gene interaction effects. Even though the asthma phenotype is relatively homogenous on Tristan da Cunha, the fact that more than one of the seven founders that populated the island after 1812 were asthmatic (Levack and Levack, 2013) suggests that the disorder manifested by the islanders is influenced by complex inheritance.

The Tristan da Cunha findings (Thompson et al., 2003, 2007, 2013), therefore, must be interpreted not only in the context of a founder effect (Zamel et al., 1996; Slutsky and Zamel, 1997; Chan-Yeung et al., 1999) but also in the context of the many genes that confer risk to asthma in out-bred populations: including GPCRs variants (**Table 1**).

### Out-Bred Populations

Genome Wide Association Studies have confirmed that variants of the *DENND1B* gene, located on chromosome 1q31 (Sleiman et al., 2010) and the *ORMDL3* gene, located on chromosome 17q12 (Moffatt et al., 2007) underlie asthma pathogenesis in many populations (Ferreira et al., 2011; Melén et al., 2013). Other genes including ADAM33, CCL5, CD14, DPP10, EDN1, NPSR1, GSTP1, IL12B, IL13, IL4, IL4R, PTGDR, TNF, and VDR have also been found to be commonly associated with asthma in various study populations (Michel et al., 2010). Asthma resulting from certain genotypes has shown distinct responses to drug treatment in some instances (Berce et al., 2013). Differentiating the genetic basis of a heterogeneous disease such as asthma, therefore, creates the opportunity to tailor specific therapeutic regimens to the resulting endophenotype (Rana et al., 2001; Milligan, 2002; Thompson et al., 2012, 2014a,c).

G protein-coupled receptors are the primary drug targets for drug interventions into many forms of asthma. Many advances in therapeutics have resulted from the identification of GPCR variants that are associated with disease. Polymorphisms of the β_2_-adrenergic receptor (*ADRB2*) gene may be among the most clinically relevant with respect to drug response (Thonkham et al., 2015). Like the subsequent study of many GPCR variants, homology with the prototypical GPCR, rhodopsin (Lefkowitz, 2004), was used to predict the functional significance of sequence alterations. Many of these studies were later evaluated *in vitro* (Rana et al., 2001; Lefkowitz, 2004).

Early studies showed that β_2_-adrenergic receptor variants with altered pharmacological response are more common in patients with asthma (Taylor and Kennedy, 2001, 2002; Taylor et al., 2005; Hawkins et al., 2008) as well as congestive heart failure (Thompson et al., 2014a). More recent studies have linked polymorphisms of *ADRB2* to altered drug response (Basu et al., 2009; Mathias et al., 2010). Asthma treatment with albuterol, which is targeted to ADRB2, is often associated with increased hypersensitivity (Reihsaus et al., 1993; Dewar et al., 1997; Liggett, 1997; Drysdale et al., 2000; Israel et al., 2000; Littlejohn et al., 2002). Work is currently underway to identify ADRB2 polymorphisms in populations, such as the residents of Grenada, where 30% of the children are reported to have asthma according to a modified ISAAC survey (Pearce et al., 2007; Thonkham et al., 2015).

Other candidate gene studies have identified GPCR gene variants that are associated with asthma phenotypes from various populations. *In vivo* and *in vitro* studies have determined functional roles for many GPCR variants (Rana et al., 2001), but evaluation of their physiological role in disease remains a challenge (Thompson et al., 2014b). In addition to CYSLTR1 and CYSLTR2 receptor polymorphisms (**Tables 2** and **3**), GPCR variants of significance to asthma pharmacogenetics are presented in **Table 1**. These receptors include the endothelin-1 receptor type A (*EDNRA*; Mao et al., 1999; Michel et al., 2010), prostaglandin D_2_ (*PTGDR2*; Cookson and Moffatt, 2004; Oguma et al., 2004), thromboxane A_2_ (*TBXA2R*; Unoki et al., 2000; Kim et al., 2005) and chemokine [C-C motif] receptor 5 (*CCR5*) receptors (Hall et al., 1999; Srivastava et al., 2003; Agrawal and Bharadwaj, 2005).

## Leukotriene Modifier (LTM) Pharmacogenetics

The cysteinyl leukotriene ligands (CysLT), including leukotriene C4 (LTC4), D4 (LTD4) and E4 (LTE4), are powerful bronchoconstrictors and pro-inflammatory mediators of atopic asthma (Samuelsson, 1983; Bisgaard, 2001). They are released by mast cells and macrophages during asthma attacks (Severien et al., 2000). The severity of an asthma attack can be influenced by the cellular concentrations of these CysLTs (Maekawa et al., 2002). These ligands regulate human airway contractions upon binding to the CysLT receptors (Metters, 1995; Takasaki et al., 2000; Figueroa et al., 2001; Kormann et al., 2005).

Polymorphic variability is found within many of the genes involved with the synthesis and signaling pathways of CysLTs. The influence of these variations to CysLT biosynthesis enzyme activity, CysLT signaling and asthma severity will be reviewed below. It is important, however, to distinguish between the effect of functional variants of risk alleles for atopy or asthma from pharmacogenetic variants that primarily influence the absorption or response to LTMs. Pharmacogenetic variants which also confer susceptibility to disease will be discussed in Section “Cysteinyl Leukotriene Receptor Polymorphisms.”

Many loci implicated in LTM pharmacogenetics, however, encode genes that are independent of CysLT-related pathways. For example, rs12422149, a common c.935G > A polymorphism in the Solute Carrier organic anion transporting polypeptide (OATP) 2B1 (SLCO2B1) was associated with absorption of and response to montelukast in humans. In particular, *in vitro* studies have shown that citrus juice may reduce the permeability of montelukast. Moreover, pharmacokinetic studies of montelukast co-ingested with citrus juice suggest that co-ingestion resulted in genotype specific alterations in absorption. For example, A/G heterozygotes were demonstrated to have reduced absorption relative to G/G homozygotes, independent of treatment or combined treatments (Mougey et al., 2011).

Increasingly, loci of pharmacogenetics significance have been identified by GWAS. These data underscore the fact that many loci that alter the efficacy of LTMs pharmaceuticals are not directly part of the CysLT pathway. Seminal studies by Dahlin et al. examined patient response to montelukast and the 5-LO inhibitor zileuton, over 8 weeks and (Dahlin et al., 2015) and 12 weeks (Dahlin et al., 2016), respectively, using DNA and phenotypic information from 526 and 217 people enrolled in placebo-controlled trials. This group based their initial analysis on the genome-wide genotype and phenotypic data from American Lung Association’s Asthma Clinical Research Center (ALA-ACRC) cohorts. These results showed that while rs6475448 [located in the myeloid/lymphoid or mixed-lineage leukemia (*MLLT3*) gene] was associated with improved montelukast response over an 8-week treatment period (Dahlin et al., 2015), rs12436663 [located in the Mitochondrial Ribonuclease P Protein 3 Precursor (*MRPP3*) gene, encoding a protein critical to the cleavage of tRNA molecules], was associated with worsened zileuton response over a 12-week period (Dahlin et al., 2016). In both studies, the change in Forced Expiratory Volume (ΔFEV) from baseline in response to drug was used as a measure of treatment response (Dahlin et al., 2015, 2016). Furthermore, the gene-environment (G × E) GWAS model evaluated in the 12-week study identified an association between rs517020 (located in the glycosyltransferase 1 domain containing 1 (*GLT1D1*) gene) with worsening responses to both montelukast and zileuton (Dahlin et al., 2016). These findings implicate loci in LTM response that, by contrast with leukotriene candidate genes, are not intuitively related to LTM therapeutic responsiveness (Dahlin et al., 2015, 2016).

Next, we will review the polymorphisms in the genes encoding portions of the CysLT pathway implicated in asthma pathogenesis and treatment (**Figure 1**) before discussing the receptor polymorphisms *per se*.

## Leukotriene Synthesis

Leukotrienes are fatty acid-derived mediators which are divided in two main biologically active subgroups, LTB4 and the cysteine-conjugated leukotrienes (LTC4, LTD4, and LTE4). The arachidonate 5-lipoxygenase (ALOX5) is required for the synthesis of the unstable epoxide precursor LTA4 (**Figure 1**), on which acts the leukotriene A4 hydrolase (LTA4H) to originate the proinflammatory chemoattractant LTB4 or the leukotriene C4 synthase (LTC4S) to conjugate the tripeptide glutathione and form LTC4; LTD4 and LTE4 are then formed by subsequent removal of the terminal amino acid of the tripeptide. The genes which encode for the leukotriene synthesis enzymes all have polymorphisms that have been investigated in asthma endophenotypes (Capra et al., 2007; Tantisira and Drazen, 2009).

### ALOX5 Polymorphism

The polymorphic region of the ALOX5 gene contains a functionally important variable number tandem repeat (VNTR) sequence (rs59439148). The VNTR of the SP1-binding motif of the ALOX5 promoter (In et al., 1997) is likely to determine the expression levels of ALOX5 (In et al., 1997; Silverman et al., 1998). An early study found that patients with no wild type (WT) allele at the ALOX5 promoter locus have a diminished response to treatment with the 5-LO inhibitor, ABT-761, which targets ALOX5 (Drazen et al., 1999b). Though this polymorphism may not predict asthma risk (Sayers et al., 2003), it may be a significant determinant of the leukotriene burden associated with bronchoconstriction (Holgate et al., 1996; Drazen et al., 1999a). More recently, ALOX5 polymorphisms have been associated with increased cysteinyl leukotriene production as well as reduced lung function in children with poorly controlled asthma (Mougey et al., 2013). In this study, 270 children, 6- to 17-years old, with poorly controlled asthma were enrolled in a 6-month clinical trial. A smaller cohort of 137 patients was examined in order to test the association of the ALOX5 promoter with asthma outcomes using both additive and recessive genetic models. Mougey et al. reported that between 14.8% (40/270) and 28% (38/135) of African Americans were homozygous for variant alleles of rs59439148. These results suggest that the ALOX5 promoter contributes to increased urinary LTE4 levels, reduced lung function and potentially worse asthma control. ALOX5 promoter variants may, therefore, be a risk factor for worse asthma outcomes.

The ALOX5 variants that confer susceptibility to asthma, therefore, may be of pharmacogenetic significance if an inadequate quantity of ALOX5 protein is available as a drug target (Lima et al., 2006; Telleria et al., 2008; Tantisira et al., 2009). Evidence that g.20C > T (rs4987105) and g.213411G > A (rs4986832) are associated with improved forced expiratory volume (FEV1) in response to montelukast, suggests the clinical relevance of the ALOX5 polymorphisms (Klotsman et al., 2007). These studies suggest that the 15% or so of individuals who carry these SNPs may have a heightened response to montelukast that reflects their endogenously excessive levels of CysLTs.

### LTC4S Polymorphism

There is evidence that the gene encoding LTC4S, the enzyme that adducts glutathione at the C-6 position of the arachidonic acid backbone (Lam and Austen, 2000), is also polymorphic. The promoter of the LTC4S gene contains a -444A > C SNP which is transcriptionally active. However, the activity of this polymorphic promoter is reported to cause alterations in CysLT production which are associated with severe asthma (Sampson et al., 2000; Kedda et al., 2004). This relationship has been confirmed by meta-analysis (Zhang et al., 2012).

The variant may be of pharmacogenetic significance. Subjects who were homozygous for the WT LTC4S -444A allele had a lower response to the CYSLTR1 antagonist zafirlukast, compared with CC or CA genotypes (Sampson et al., 2000). Two recent studies report the profound physiological and functional significance of this polymorphism. The first study found that, compared with corn oil, a combination of borage and echium seed oils may improve airflow obstruction in mild to moderate asthmatics who carry the variant allele in the LTC4S gene (A-444C; Kazani et al., 2014). The second group that investigated urinary leukotrienes reported that leukotriene E4 levels may be higher in patients with the LTC4 synthase -444 A/C polymorphism (Kadry et al., 2014).

The efficacy of LTMs such as Montelukast are influenced my many polymorphisms in addition to -444A/C *LTC4* polymorphisms. For example, the -441T/C polymorphism of the prostaglandin D2 receptor (*PTGDR*) gene is associated with treatment responsiveness to montelukast. In particular, when LTA release was examined by exercise challenge in 100 asthmatic children prescribed 5 mg/kg Montelukast, it was found that minor alleles of the *PTGDR* -441T/C and *LTC4S* -444A/C polymorphisms were associated with eosinophil count in atopic asthmatic children. While the *LTC4S* -444A/C and *PTGDR* -441T/C were not associated with the susceptibility for asthma, there was a significant association between responsiveness to montelukast and the *PTGDR* polymorphism. These pharmacogenetic data suggest that therapies that target the PTGDR may be useful for modulating the responsiveness to LTRAs (Kang et al., 2011).

These observations suggest that the genes encoding the CysLT pathway (**Figure 1**), may contribute additively or synergistically to altered drug responses or asthma endophenotypes. This phenomenon was previously suggested by Park et al. (2005). This group reported that the *LTC4S* -444A > C [c.-444A > C] gene polymorphism, in conjunction with the rare c.-819T > G, c.2078C > T or c.2534A > G alleles of the *CYSLTR1* gene, was associated with aspirin intolerant asthma (Park et al., 2005). Al-Shemari et al. (2008) initially showed that SNPs of the *ALOX5, CYSLTR1*, and *ALOX5* genes were found to be associated with allergic rhinosinusitis. However, the association was diminished upon correction for multiple testing. This may have resulted because the study lacked the power to detect polymorphisms conferring a relative risk of 2.0 or less to the phenotype: reflecting the fact that individual risk factors for complex disease can be quite small (Al-Shemari et al., 2008). However, while an association of the *LTC4S* -1072G/A, rs377694, and rs730012A/C haplotype with asthma was reported, no evidence of an interaction with the *CYSLTR1* gene was identified (Kumar et al., 2012).

Study of genetic variability in the genes conferring response to LTMs, including those encoding the CysLT pathway (**Figure 1**), therefore, should be subjected to GWAS analysis since this method is well-suited to assessing the addictive or multiplicative contribution of genes complex traits.

## CYSTEINYL Leukotriene Receptor Polymorphisms

There are two distinct genes which encode the two CysLT receptors: the cysteinyl leukotriene 1 (*CYSLTR1*) and 2 (*CYSLTR2*) receptors genes. These two receptor proteins exhibit variable tissue expression, however, both are expressed in lung macrophages and in airway smooth muscle (ASM; Metters, 1995; Lynch et al., 1999; Heise et al., 2000; Takasaki et al., 2000; Figueroa et al., 2001; Mita et al., 2001). Knock-out mouse studies have given insight into the pathobiology mediated by the CysLTs (Kanaoka et al., 2001). *CYSLTR2* was revealed to have a major role in chronic inflammation associated with fibrosis (Beller et al., 2004a). By contrast, *CYSLTR1* was found to be associated with acute constriction of smooth muscle and anti-inflammatory counteraction of chronic injury (Beller et al., 2004b). Biochemical studies have also shown that these two receptors are functionally distinct. Although, CYSLTR1 has its highest affinity for LTD4 (Lynch et al., 1999; Woszczek et al., 2005), CYSLTR2 binds LTC4 and LTD4 with equal affinity (Heise et al., 2000; Maekawa et al., 2001); a phenomenon that may define the specificity of the acute and chronic activity of the CysLTs.

As with other GPCR genes, variants of the cysteinyl leukotriene receptors (CysLT) include single nucleotide polymorphisms and insertion or deletions that alter GPCR expression or function (Balasubramanian et al., 2005). Early evidence of the possible significance of CysLT receptor variability was observed in mice: in which the *CYSLTR1* transcript has been shown to undergo alternative splicing that alters the function of the protein (Maekawa et al., 2001). From 2001 to the present, a number of groups have been investigating the contribution of variability of the two CysLT receptors to atopic asthma and response to pharmacological interventions. Selected CysLT receptor polymorphisms are presented in **Tables 2** and **3**.

Human polymorphisms, including coding variants as well as has functional promotor haplotypes that either increase or decrease the expression of either receptor are presented in **Tables 2** and **3**. The epidemiology and *in vitro* studies discussed below suggest that functional receptor variants are associated with asthma endophenotypes and altered pharmacological response once environmental factors such as smoking were controlled for (Thompson et al., 2014a). The disturbances include the alteration of: ligand binding; G-protein coupling; dimerization; desensitization; receptor trafficking to the cell surface as well as receptor mRNA expression (Rana et al., 2001; Thompson et al., 2014a).

Early studies of the effect of amino acid substitutions in the CYSLTR1 and CYSLTR2 receptor genes in atopic asthma were based on a personalized medicine hypothesis (Thompson et al., 2005a, 2006, 2014b; Capra et al., 2007). The LTM class of drugs has been found to be ineffective in greater than 20% of patients (Noonan et al., 1998). We originally hypothesized that genetic polymorphisms influenced the efficacy of drugs that act at the CYSLTR1 receptor, such as the high-affinity antagonist ligands (e.g., montelukast, pranlukast, zafirlukast; Reiss et al., 1996; Grossman et al., 1997; Suissa et al., 1997; Obase et al., 2001). Further, we proposed that even though the CYSLTR2 receptor is not a current drug target, knowledge of the polymorphisms of this potential drug target maybe clinically valuable to refractory patients in the future.

We discuss these findings in the context of the body of cysteinyl leukotriene receptor pharmacogenetics that has since been established. Selected CYSLTR1 and CYSLTR2 polymorphisms are presented in **Tables 2** and **3**.

### Cysteinyl Leukotriene 2 Receptor Polymorphisms

**FIGURE 2:**
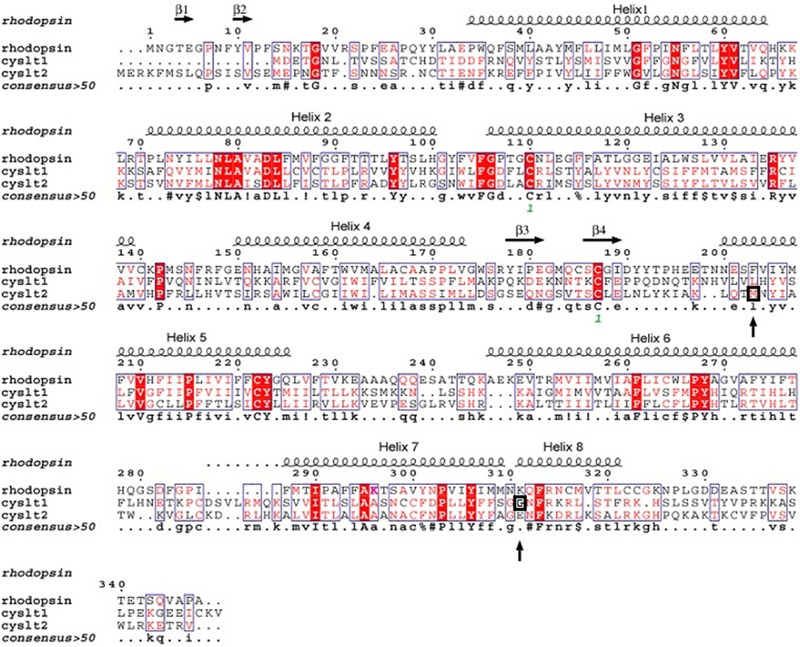
**Alignment of the protein structure of the cysteinyl leukotriene 1 (CYSLTR1) and 2 (CYSLTR2) receptors in relation to rhodopsin.** The amino acids conserved between these family A receptors are shown. The consensus is greater than 50%. These data formed the basis of the model predicting the CYSLTR1 and CYSLTR2 transmembrane domains (helices 1–7), the four β-sheets, and the putative cysteinyl leukotriene-binding domain. The amino acid variants that are associated with atopy or asthma, the G300S CYSLTR1variant, and the M201V CYSLTR2 variant are each boxed and noted with arrows (adapted from Thompson et al., 2014a).

**FIGURE 3:**
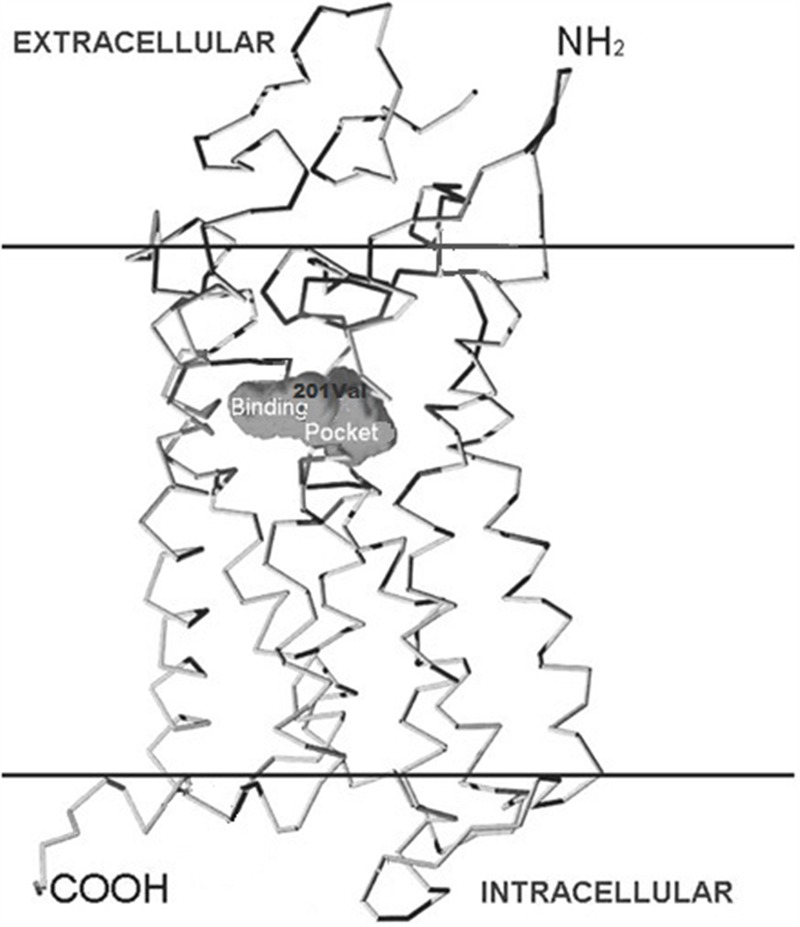
**Structure of the cysteinyl leukotriene 2 (CYSLTR2) receptor and variants.** The positions of the transmembrane (TM)-spanning domains of the CysLT receptor, the putative binding pocket, and the Met201Val amino acid substitution are shown in relation to the cutaway plasma membrane.

Four independent groups (Thompson et al., 2003, 2013) have identified significant associations between the CYSLTR2 gene loci and atopy (Fukai et al., 2004; Pillai et al., 2004; Park et al., 2005; Thompson et al., 2013). The amino acid alignment (**Figure 2**) shows the location of CYSLTR2 variant with respect to the transmembrane (TM) domains. The CYSLTR2 receptor is shown schematically in **Figure 3**.

The 610 A > G variant which encodes the Met201Val CYSLTR2 receptor variant was originally identified in the Tristan da Cunha population has also been reported by Pillai et al. (2004). A less remarkable Ser236Leu variant, which was originally identified in the Tristan population (Thompson et al., 2003), may be present in as many as 10% of persons of African descent according to the Exome Aggregation Consortium, ExAC^1^. Though the Ser236Leu variant has not been examined in asthmatics of African origin, it has been seen compounded with Met201Val. This may be important for Tristanian asthmatics identified to be compound heterozygous for the Met201Val and Ser236Leu variants of CYSLTR2.

The Met201Val variant that was found to be associated with atopic asthma on Tristan da Cunha (**Table 3**) has subsequently been reported to have frequency of between 2.6% in the general population according to the Exome Aggregation Consortium (ExAc)^1^. The essentially inactivating character of the Met201Val the polymorphism (Thompson et al., 2003), to be discussed in below, has been confirmed by the group of Pillai et al. (2004) Their data show that Met201Val is associated with asthma phenotypes in a large population genetics study. Definitive studies using SNPs that saturate *CYSLTR2* may be necessary before these findings are placed in context, however, since association studies with non-coding SNPs or haplotypes built from only a few SNPs (Kato et al., 2011; Kumar et al., 2012) are unlikely to be definitive.

### Cysteinyl Leukotriene 1 Receptor Variants

The presence of SNPs in both the *CYSLTR1* and *CYSLTR2* genes may increase the risk for atopic asthma (**Tables 2** and **3**). Since the CYSLTR1is the target for anti-asthma drugs such as montelukast, the *CYSLTR1* SNPs may provide an opportunity to examine the amino acid residues necessary to the structure-activity relationships that define drug action. The *CYSLTR1* gene, located at Xq13-21.1, has been examined in atopic asthma (**Table 2**). *CYSLTR1* variants, such as the 927 T > C variant (rs320995), have been associated with asthma (Hong et al., 2009) in out-bred populations. While this SNP was also noted in the Tristan da Cunha population (Thompson et al., 2013), it has not been replicated in all studies of atopic asthma (Kadry et al., 2014). A nucleotide substitution at 898G > A resulting, *in vitro*, in a functional Gly300Ser substitution (Thompson et al., 2007) is discussed below.

Taken together, many studies suggest that transcriptionally active *CYSLTR1* variants influence the onset and severity of asthma associated endophenotypes such as atopy (Sokolowska et al., 2009). The significance of *CYSLTR1* variants can be interpreted in the context of a large study of asthmatics born in Great Britain during 1958. This study, however, did not identify coding variants associated with atopy or asthma (Duroudier et al., 2009). Among those variants examined *in vitro*, only the 206Ser variant has been found by the Exome Aggregation Consortium^1^. Our ongoing GWAS study of Tristan da Cunha will ultimately resolve the questions raised by the pioneering work we undertook in this population.

### Co-inheritance of CYSLTR1 and CYSLTR2 Variants

We can hypothesize that epistasis involving inheritance of variants of both receptors contributes to the phenotype. The putative co-inheritance of *CYSLTR1* and *CYSLTR2* variants suggest a mode of asthma inheritance that is the result of epistasis. Proof of principal may be found in data showing that variants of two genes encoding other GPCR pathway proteins appear to be implicated in asthma pathogenesis. For example, it is likely that an interaction between variant forms of *NPSR1* with variants of the retinoid receptor-related orphan receptor alpha (*RORA*) nuclear repressor gene may contribute to a variety of asthma phenotypes (Acevedo et al., 2013). Similarly, the contribution of *CYSLTR1* and *CYSLTR2* alleles may contribute additively or synergistically to asthma in association with other risk alleles and environmental factors.

## Pharmacology of CYSLTR1 and CYSLTR2 Receptor Variants

The associations of the *CYSLTR1* and *CYSLTR2* genes with atopy and asthma provided the rationale for studying the functional consequences of these variants with respect to agonist and their possible contribution to the atopy phenotype (Thompson et al., 2003, 2007, 2013). By contrast, a putative high affinity LTE4 receptor (Kanaoka et al., 2013), GPR99 (also known as an oxoglutarate receptor) may not be involved since the actions of LTD_4_ can account for the phenotype at the cognate CYSLTR1 and CYSLTR2 receptors. The CysLT receptor CYSLTR2 variants are discussed in the context of their potential to influence LTM response.

### Functional Variants of CYSLTR2

**FIGURE 4:**
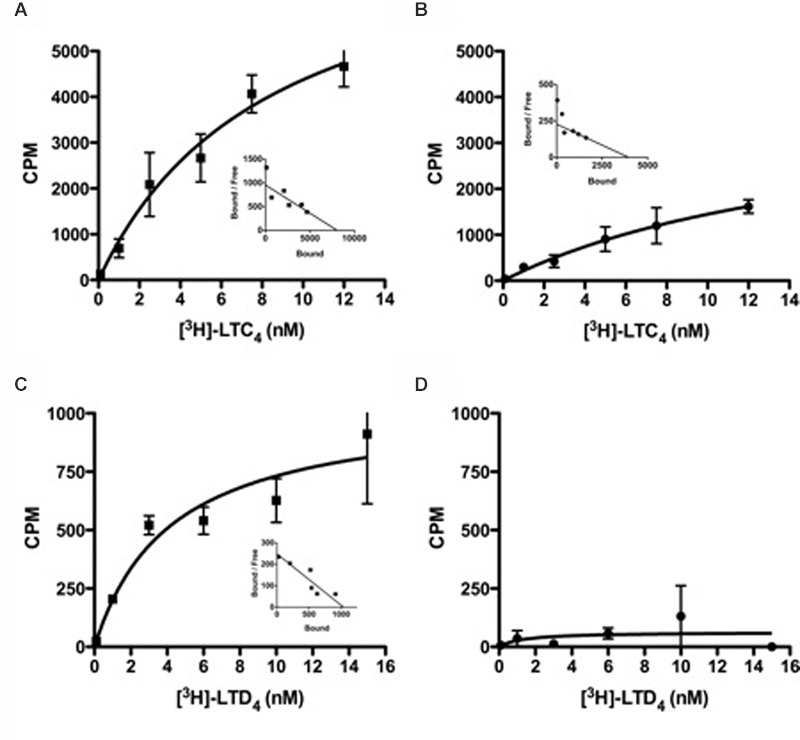
**Radioligand binding studies of LTC_4_ or LTD_4_ with CYSLTR2 WT or CYSLTR2 Met201Val cells. (A,B)** Saturation binding isotherms for [^3^H]LTC_4_ to CYSLTR2 WT **(A)** or CYSLTR2 Met201Val **(B)**. Radioligand binding assay was performed with increasing amounts of [^3^H]LTC_4_ (0.1–12 nM) in the presence or absence of unlabeled ligand. **(C,D)** Saturation binding isotherms for [^3^H]LTD_4_ to CYSLTR2 WT **(C)** or CYSLTR2 Met201Val **(D)**. Radioligand binding assay was performed with increasing amounts of [^3^H]LTD_4_ (0.1–15 nM) in the presence or absence of unlabeled ligand. The calculated specific binding was analyzed by non-linear transformation using Prism (Graph Pad Software, Inc.) to generate *K*_d_ and *B*_max_ values. Saturation curves are representative of four independent experiments. Insets: Scatchard representation of the specific binding isotherm (adapted from Brochu-Bourque et al., 2011).

**FIGURE 5:**
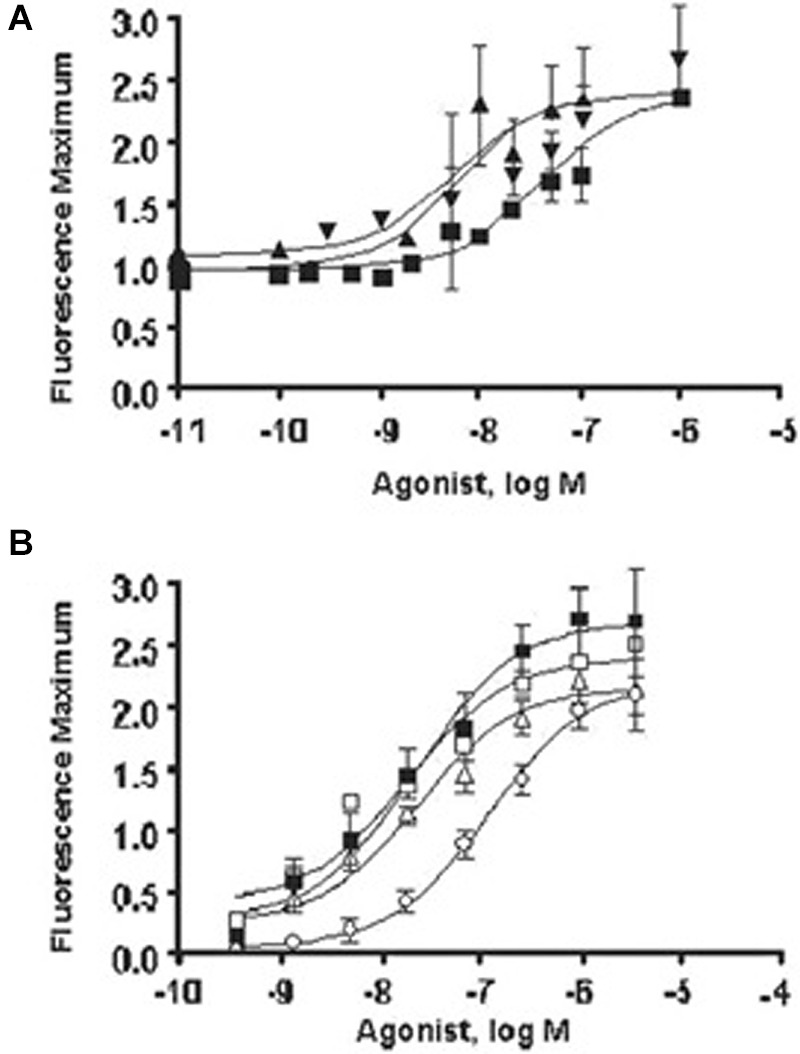
**Summary of the *in vitro* effects of Gly300Ser cysteinyl leukotriene 1 (CYSLTR1) receptor and Met201Val on CYSLTR2 receptor signaling compared with wild type (WT). (A)** Cysteinyl leukotriene D (LTD) concentration-response curve for CysLT receptors in transfected cells. Inositol triphosphate (InsP) generation assay of the variants and WT forms of the CYSLTR1 receptor. both 300Ser and 206Ser variants’ EC50 were significantly different from WT. The concentrations of LTD required to produce the InsP effect were much higher than those used in the [Ca^2+^] assay shown in **(B)**, in which calcium flux was assayed for the variants and WT and variant forms of the CYSLTR1 receptor challenged with LTD. The resulting changes in intracellular calcium concentrations were measured as fluorescence maximum. For LTD4, the Met201Val variant (○) had a significantly greater EC50 compared to WT (■), while the Ser236Leu(Δ) and Ala293Gly/Arg316Lys (^®^) variants were not different. However, the Ala293Gly/Arg316Lys variant showed decreased efficacy (V; adapted from Thompson et al., 2014a).

**FIGURE 6:**
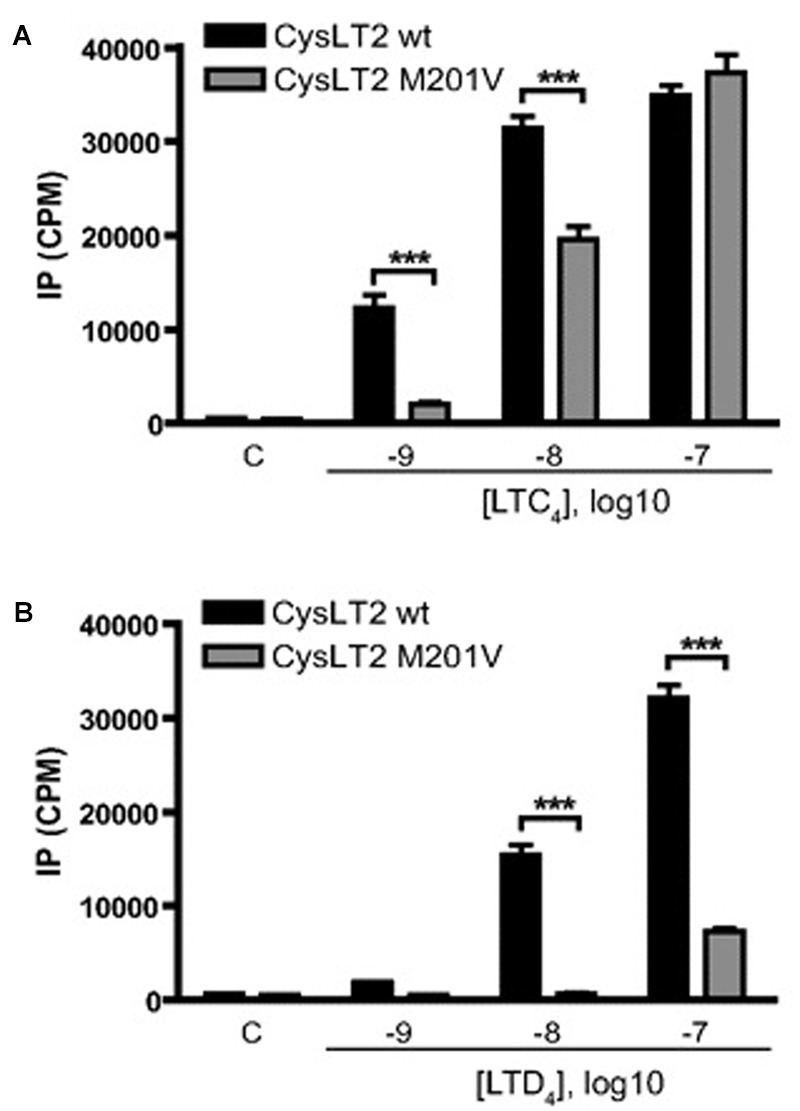
**Effect of CysLTs on IP production by CYSLTR2 WT (black bars) or CYSLTR2 Met201Val (gray bars) cells.** Cells were labeled with [^3^H]myo-inositol for 16 h, and then stimulated for 45 min with EtOH and LTC_4_ **(A)** or LTD_4_ **(B)** at the indicated concentrations before measurement of radioactivity. Results are presented as cpm of inositol phosphate production. Data are expressed as mean ± SE of 15 **(A)** or 9 **(B)** independent experiments. Lane C, control using EtOH (100 nM). ^∗∗∗^*p* < 0.001, using two-way ANOVA with Bonferroni post-tests (adapted from Brochu-Bourque et al., 2011).

The 601 A > G variant that is associated with atopic asthma in both the Tristan da Cunha (Thompson et al., 2003) and European populations (Pillai et al., 2004) encodes the Met201Val variant of transmembrane domain five (TMD5; **Figure 3**). GPCR models suggest this variant creates a mildly deleterious hypomorphic protein (Bromberg and Rost, 2007; Kazius et al., 2008; Bromberg et al., 2009; Vroling et al., 2011; Rana et al., 2012; Thompson et al., 2014a). Our work has recently confirmed the variant to be hypomorphic (Brochu-Bourque et al., 2011). The CYSLTR2 inactivating mutation results in reduced ligand binding, Ca^2+^ flux (**Figures 4D** and **5B**) and inositol phosphate (IP) generation (**Figure 6**; Thompson et al., 2003; Pillai et al., 2004; Brochu-Bourque et al., 2011).

In general, the effects of Met201Val were more pronounced with respect to LTD_4_ compared with LTC_4_. This effect is compounded by deficits in ligand binding that were again more significant for LTD_4_ than LTC_4_ (**Figure 4**). The fact that LTD_4_ release is more commonly associated with asthma severity, may explain why this variant is clinically relevant. The fact that the CYSLTR2 variant is both relatively common and associated with asthma in out-bred populations suggests that the variant is of relevance to the general population.

### Functional Variants of CYSLTR1

**FIGURE 7:**
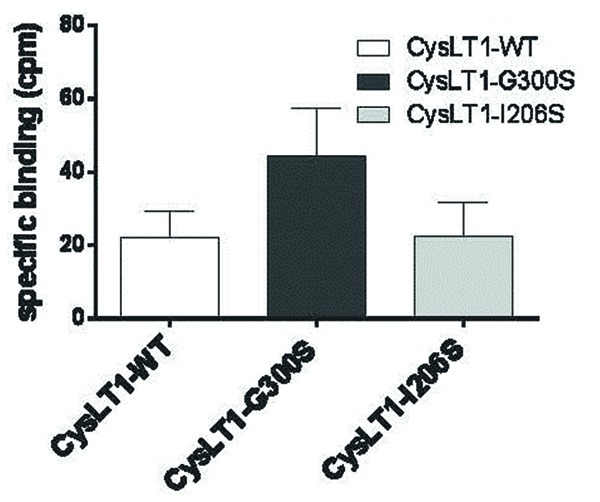
**Binding of LTD4 to the CYSLTR1-WT receptor and its variants in transiently transfected HEK293 cells.** Forty-eight hours post-transfection, 3H-LTD4 was added to the cells for 1 h, in the presence or absence of non-radioactive LTD4. Following several washes, the radioactivity was measured in a beta-counter (*n* = 3). Specific binding was calculated by subtracting the non-specific binding (in presence of competition from radioactive LTD4) from the total binding measured (Yaddaden et al., 2016).

**FIGURE 8:**
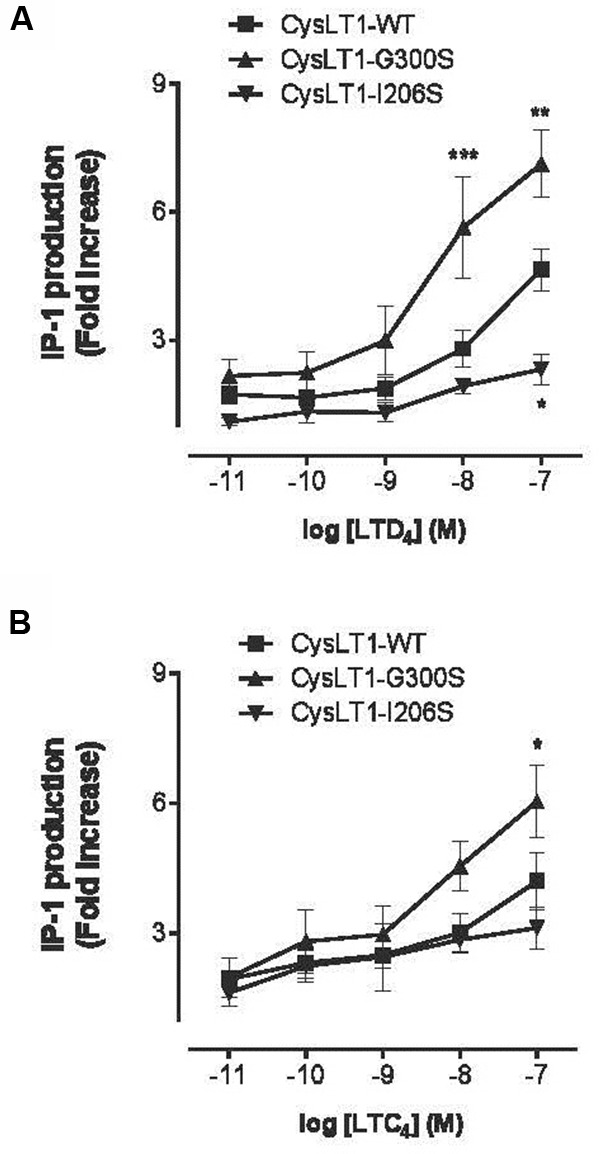
**Inositol phosphate production in CYSLTR1-transfected cells.** Forty-eight hours post-transfection, cells were stimulated for 30 min with LTD4 or LTC4 at 0.01–100 nM. IP-1 production was measured using an antibody against IP-1 and a fluorescent FRET IP-1 conjugate competitor. **(A)** IP-1 production by CYSLTR1-transfected HEK-293 cells in response to LTD4 **(B)** and to LTC4. Two-way ANOVA analysis; *n* = 6; ^∗^*p* < 0.05, ^∗∗^*p* < 0.01, ^∗∗∗^*p* < 0.001 (adapted from Yaddaden et al., 2016).

Since the CYSLTR1 Gly300Ser variant has not been reported in the general population, our functional analysis of the Gly300Ser variant will be especially informative if the CYSLTR1 gene is implicated in disease pathogenesis. Our *in vitro* study of the Gly300Ser variant (**Figures 7** and **8**; Yaddaden et al., 2016) provided among the first structural insights into CysLT receptors using site directed mutagenesis. This represents a significant advance in understanding the relatively under-studied GPCR receptors for the cysteinyl leukotrienes.

When the engineered Gly300Ser variant receptor was expressed it in COS-7 cells, the variant was more sensitive to the LTD_4_ agonist (**Figures 7** and **8**). Binding properties of the variant (measured by B_max_ and K_d_) indicates that the CYSLTR1 variant has higher affinity for ligand than the WT (**Figure 7**) by comparison with the CYSLTR2 polymorphism that has less affinity for ligand than the WT (**Figure 4**). By contrast, the CYSLTR1 206Ser variant, originally identified in the Tristan da Cunha population, and later found at low frequencies in atopics, asthmatics and control subjects throughout the world, was not found to be functional (Thompson et al., 2007; Yaddaden et al., 2016).

**FIGURE 9:**
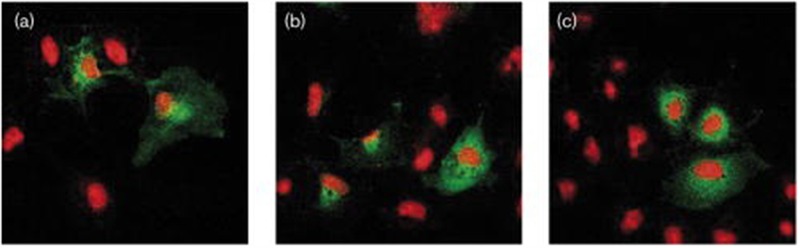
**Confocal microscopy imaging of cysteinyl-leukotriene 1 (CYSLTR1) WT and variant receptors in transiently transfected cells.** Cells were fixed and immunofluorescently stained with anti-CYSLTR1R antibody (green) followed by Alexa 488 goat anti-rabbit antibody (red) as described under Methods section. Superimposed images of immunoreactivity of **(a)** WT CYSLTR1 receptor, **(b)** Gly300Ser CYSLTR1 receptor variant or **(c)** Ile206Ser CYSLTR1 receptor variant, in basal condition. One representative experiment is shown (adapted from Thompson et al., 2007).

In fact, while ligand binding (**Figure 7**), IP generation (**Figure 8**) and Ca^2+^ flux (**Figure 5A**) was reduced compared to CYSLTR1 WT, the expression of the CYSLTR1 Gly300Ser variant receptors (**Figure 9**) and their desensitization in response to agonist was found to be normal (Thompson et al., 2007). These data suggest that CYSLTR1 CYSLTR1 antagonists, such as montelukast, may correct a putative elevation in CYSLTR1 signaling in atopic asthma.

The relative location of variants of the receptors is shown in an alignment of each CysLT receptor (**Figure 2**) that was constructed with respect to the transmembrane-spanning and the putative binding pocket of the receptors. The data suggests that the putative CysLT binding site is partially determined by the integrity of the transmembrane domains. The abnormal but opposite pharmacology of variants of the receptors, causing increased potency of LTD_4_ at the Gly300Ser CYSLTR1 receptor variant (located in the intracellular portion of TMD7) and decreased potency of LTD_4_ at the Met201Val CYSLTR2 receptor variant (located in the extracellular portion of TMD5) contributes important *in vitro* data on the structural significance of the specific amino acid residues in these GPCRs.

### Putative Interaction between the CYSLTR1 and CYSLTR2 Receptors

It has been proposed that macrophages and eosinophils attenuate the strength of CysLT-mediated signaling by limiting the formation of CYSLTR1 receptor homodimers and/or controlling their surface expression. With respect to MAP Kinase signals, it seems possible that WT CYSLTR2 receptors interact with WT CYSLTR1 receptors to inhibit functional responses of WT CYSLTR1 receptors. In mast cells, ligand activation of the WT CYSLTR2 receptor results in reduced signaling from ligand activated CYSLTR1 WT receptors. Although, the CYSLTR1 and CYSLTR2 receptor interaction is best modeled with respect to mitogenic signals (Jiang et al., 2007), it is possible that more rapid processes such as IP generation or calcium flux may be analogous. We hypothesize that a CYSLTR1/CYSLTR2 “counterbalance” may regulate CysLT-dependent functions in immune and inflammatory responses. This balance may be disrupted by the Met201Val variant of CYSLTR2 if the LTD_4_ dependent signal is necessary to balance the LTD4 ligand-dependent CYSLTR1 signal. This effect may be amplified in people who also carry a *CYSLTR1* variant.

The CYSLTR2 receptor may be important to the pharmacology of CYSLTR1 pharmaceuticals if, like many GPCRs, these receptors form functional heterodimers with unique pharmacological properties. Co-expression of variant receptors may alter CysLT signaling. While evidence for a functional interaction between CYSLTR1 and CYSLTR2 receptors in mast cells seems likely (Jiang et al., 2007), the functional consequence of putative heterodimers, or other functional interactions formed between variants, remains to be fully demonstrated. The fact that deletion of either the CYSLTR1 or CYSLTR2 receptors totally eliminates CysLT-mediated cutaneous edema (Lynch et al., 1999), however, suggests that a CYSLTR1/CYSLTR2 interaction may regulate some responses to CysLT mediators. It may be possible to test the hypothesis that CYSLTR2 negatively regulates CYSLTR1 signaling by examining the signaling consequences of co-expressing CYSLTR1 variants with the 201Val CYSLTR2 variant.

## Conclusion

Central to this narrative is a discussion of how variability in the genes encoding the proteins essential for CysLT signaling may influence risk for inflammation that is associated with asthma, cognizant of the fact that variability these genes may also increase susceptibility to asthma independent of their potential LTM pharmacogenetic relevance. Asthma susceptibility loci, which confer risk for the complex respiratory traits that lead to asthma, and pharmacogenetic loci, which confer the traits that define the responsiveness of an individual to pharmaceutical therapy, are presented in the context of the linkage analysis, candidate gene studies and/or GWAS approaches used to identify them. In most cases, these loci can be modeled as independent complex traits with both genetic and environmental (GXE) contributions. Therefore, we review the relative contribution of genes in the CysLT pathway to both the etiology of asthma itself and a person’s responsiveness to LTM drugs.

Common genetic variants (e.g., SNPs) typically contribute effects of as little as 1% to a complex phenotype described in a given population. As a result, GWAS can be used to confirm the significance of the cysteinyl leukotriene gene variants to atopic asthma on Tristan da Cunha. While it is possible that affected Tristanians may inherit a variety of other genetic risk factors for atopy and/or asthma, including potential *ETS-2* and *ETS-3* variability (Baron et al., 2002; Silverman et al., 2002; Wu et al., 2008), we present evidence that suggests that the *CYSLTR2* 601 A > G variant is associated with atopic asthma. The contribution of other known atopy variants, such as those in the *DENND1B* gene, located on chromosome 1q31 (Sleiman et al., 2010) and the *ORMDL3* gene, located on chromosome 17q12 (Moffatt et al., 2007) to the asthma phenotype is also possible. Like other asthma susceptibility loci, the *CYSLTR2* 601 A > G variant has been shown to contribute to asthma in large out-bred populations (Pillai et al., 2004).

While the *CYSLTR2* variant was originally identified in 13% of the Tristan da Cunha population (Thompson et al., 2003) it is only present in approximately 2.6% in the European population. In both instances, the variant has been associated with atopic asthma (Thompson et al., 2003; Pillai et al., 2004). A large study of *CYSLTR1* variants in asthmatics born in Great Britain during 1958 did not identify the 898 G > A variant (Duroudier et al., 2009) nor is it found in over 60,000 exomes by the Exome Aggregation Consortium^1^. The 898 G > A *CYSLTR1* is, however, among the best studied coding variants of a receptor for any LTM drug.

By contrast with asthma susceptibility loci, in most cases the pharmacogenetic determinants of drug response represent a distinct category of risk loci that may not influence disease onset directly. For example, the pharmacogenetic variability that influences LTM response can be attributable to genes that are both integral (Kang et al., 2011; Mougey et al., 2013; **Figure 1**), or lie outside (Dahlin et al., 2015, 2016) CysLT signaling pathways. In this context, CysLT receptor variants have provided insight into the role of GPCR variants as genetic risk factors for disease, altered drug response, or ADRs (see **Table 1**) and may represent reagents necessary for refined LTM drug discovery and/or personalized medical interventions (Spiegel and Weinstein, 2004). The significance of the CysLT receptor variants to both the development of asthma and LTM response, however, is best evaluated in the context of other polymorphisms that influence the phenotype. For example, the enzymes involved in the formation of the leukotrienes (**Figure 1**) often result in variable production of the CysLTs (LTC4, LTD4, and LTE4) and LTB4. The many examples discussed include, the VNTR sequences located in the Sp1-binding motif within the promotor region of the ALOX5 gene that are associated with both LTM response, leukotriene burden and bronchoconstriction (Drazen et al., 1999a), and the 444A > C SNP polymorphism in the LTC4S gene that is associated with severe asthma, leukotriene burden and altered response to the CYSLTR1 LTMs (Sampson et al., 2000; Kadry et al., 2014; Kazani et al., 2014). Genetic variability in CysLT pathway may contribute additively or synergistically to altered drug responses.

Since very large GWAS studies are needed to detect polymorphisms conferring a relative risk of 2.0 or less to the phenotype (Al-Shemari et al., 2008), results suggesting that *CTSLT1R* variants may interact with other polymorphic variants can be difficult to replicate (Kumar et al., 2012). This may reflect the fact that LTM targets can be quite diverse. For example, the fact that *PTGDR* polymorphism has been significantly associated with responsiveness to montelukast may confound earlier studies of LTM pharmacogenetics. Indeed, LTM therapies may be developed that selectively target the PTGDR since this receptor may be a useful way to modulate responsiveness to LTRA (Kang et al., 2011).

Many genes that are associated with altered efficacy of LTMs pharmaceuticals, however, are not directly part of the CysLT pathway. For example, variation in the SLCO2B1 gene has associated with altered absorption of montelukast administered in the presence of citrus. These data suggest that genetic factors influence pharmacokinestics of LTM response (Mougey et al., 2011).

GWAS studies of LTM response in people enrolled in placebo-controlled trials have also been undertaken. Dahlin et al. (2015, 2016) examined LTM response using DNA and phenotypic information from placebo-controlled trials from the American Lung Association’s Asthma Clinical Research Center (ALA-ACRC) cohorts. SNPs that both favored [rs6475448, located in the myeloid/lymphoid or mixed-lineage leukemia (MLLT3) gene; Dahlin et al., 2015], or worsened [rs12436663, located in the Mitochondrial Ribonuclease P Protein 3 Precursor (MRPP3) gene], rs517020 [located in the glycosyltransferase 1 domain containing 1 (GLT1D1) gene] LTM response have been identified (Dahlin et al., 2016). These findings implicate loci in LTM response that, by contrast with CysLT receptor genes or the biosynthesis genes (**Figure 1**), are not intuitively related to LTM therapeutic responsiveness (Dahlin et al., 2015, 2016).

As a result, it is necessary that a GWAS approach is used to confirm the significance of the putative contribution of cysteinyl leukotriene gene variants to asthma and/or drug response in the context of other asthma susceptibility genes and genes that influence LTM pharmacogenetics. This strategy might we reveal novel genotype–phenotype correlations; as we did for the melanocortin 1 receptor (MC1R) variants (Beaumont et al., 2007).

With respect to Tristan da Cunha, this analysis is likely to identify variety of other genetic risk factors for atopic asthma and/or LTM drug response. These include loci associated with asthma in other populations such as the *DENND1B* gene, located on chromosome 1q31 (Sleiman et al., 2010). Exclusion of the *ORMDL3* gene, located on chromosome 17q12 (Moffatt et al., 2007), may also be possible. In addition, loci already implicated in the Tristan phenotype such as *ETS-2* and *ETS-3* (Baron et al., 2002; Silverman et al., 2002; Wu et al., 2008) and the better documented *CYSLTR2*, can be evaluated in the context of those loci, such as *CYSLTR1*, that may have pharmacogenetic significance.

## Author Contributions

All authors listed, have made substantial, direct and intellectual contribution to the work, and approved it for publication.

## Conflict of Interest Statement

The authors declare that the research was conducted in the absence of any commercial or financial relationships that could be construed as a potential conflict of interest.

## Footnotes

1 http://exac.broadinstitute.org
